# A Real-Time Detection Method of Hg^2+^ in Drinking Water via Portable Biosensor: Using a Smartphone as a Low-Cost Micro-Spectrometer to Read the Colorimetric Signals

**DOI:** 10.3390/bios12111017

**Published:** 2022-11-14

**Authors:** Yifan Gu, Leizi Jiao, Fengjing Cao, Xinchao Liu, Yunhai Zhou, Chongshan Yang, Zhen Gao, Mengjie Zhang, Peng Lin, Yuxing Han, Daming Dong

**Affiliations:** 1 College of Electronic Engineering (College of Artificial Intelligence), South China Agricultural University, Guangzhou 510642, China; 2 National Research Center of Intelligent Equipment for Agriculture, Beijing Academy of Agriculture and Forestry Sciences, Beijing 100097, China; 3 Shenzhen International Graduate School, Tsinghua University, Shenzhen 518055, China; 4 RIOS Lab, Tsinghua University, Shenzhen 518055, China

**Keywords:** colorimetric sensor, smartphone, micro-spectrometer, gold nanoparticle, Hg^2+^ detection

## Abstract

This paper reported a real-time detection strategy for Hg^2+^ inspired by the visible spectrophotometer that used a smartphone as a low-cost micro-spectrometer. In combination with the smartphone’s camera and optical accessories, the phone’s built-in software can process the received light band image and then read out the spectral data in real time. The sensor was also used to detect gold nanoparticles with an LOD of 0.14 μM, which are widely used in colorimetric biosensors. Ultimately, a gold nanoparticles-glutathione (AuNPs-GSH) conjugate was used as a probe to detect Hg^2+^ in water with an LOD of 1.2 nM and was applied successfully to natural mineral water, pure water, tap water, and river water samples.

## 1. Introduction

The distribution of mercury pollution within the various spheres of the Earth system has been attracting long-term attention because of the substantial toxicity of mercury [1]. Hg^2+^ is one of the most stable inorganic forms of mercury and has characteristics highly hazardous to health at low concentrations [2], such as permanent damage to the human digestive system, urinary system, and nervous system. Therefore, to prevent Hg^2+^ from threatening the health of human beings and ecosystems as much as possible, it is necessary to perform qualitative and quantitative detection of the trace levels of Hg^2+^ in water.

Several well-established methods are currently available for detection of heavy metals in drinking water and in its source, including atomic absorption spectroscopy [3,4], atomic fluorescence spectroscopy [5,6], inductively-coupled plasma mass spectrometry [7,8], and gas chromatography-mass spectrometry (GC-MS) [9]. Many rapid-detection methods have been used to improve detection efficiency, including colorimetric methods based on the nucleic acids, antibodies, nanomaterials, or paper [10,11], fluorescence methods [12,13], electrochemical conduction methods [14,15], and recently-developed surface Raman-enhanced scattering methods [16]. Although these methods have been shown to provide high sensitivity and accuracy using biological and chemical reactions, there are some drawbacks that must still be addressed, such as difficulty in preparing nucleic acid aptamers or antibodies, low ligation efficiency, extended response time, complexity, and short lifetime. In contrast, colorimetric biosensors based on nanotechnology are more attractive because of their simple preparation processes, low cost, and excellent readability [17,18,19]. Gold nanoparticles (AuNPs) are favorable for colorimetric biosensing applications due to the high molar extinction coefficient, high specific surface area, and easy functionalization, leading to color changes that are related to their interparticle distances [20,21,22,23]. Additionally, the spectrophotometer is widely used in biological colorimetric sensors as an important analytical device. However, the cost of spectrophotometers for non-laboratory-based applications is high, the available software support is severely limited due to the scalability by specific permissions, and the device is strongly dependent on professional and proficient operators with regard to data processing [24]. Additionally, an adequate platform is required to place and power the spectrometer. Therefore, the development of a portable spectrophotometer is essential to reduce detection costs and increase simplicity, and smartphones have gradually emerged as an outstanding option [25,26,27].

Research trends and innovative approaches in the testing field have begun to focus more on light device weight, simplicity, and effectiveness. Over the past decade, smartphones have become indispensable personal devices for many people and are attracting considerable attention for low-cost, low-power, portable, and high-speed biosensors [28,29,30]. Among the device features, complementary metal-oxide-semiconductor (CMOS) image sensors on smartphones can convert optical signals into electrical signals [31]. With the continuous advancements in their integrated circuit design and manufacturing technology, smartphone cameras are increasingly improving sensitivity, resolution, and dynamic range with reduced power consumption [32,33]. These cameras have even been applied to smart cars, which are more demanding than smartphones [34]. These advantages enable smartphones to be used as readout tools for portable colorimetric biosensors to measure color and intensity changes effectively.

**Figure 1:**
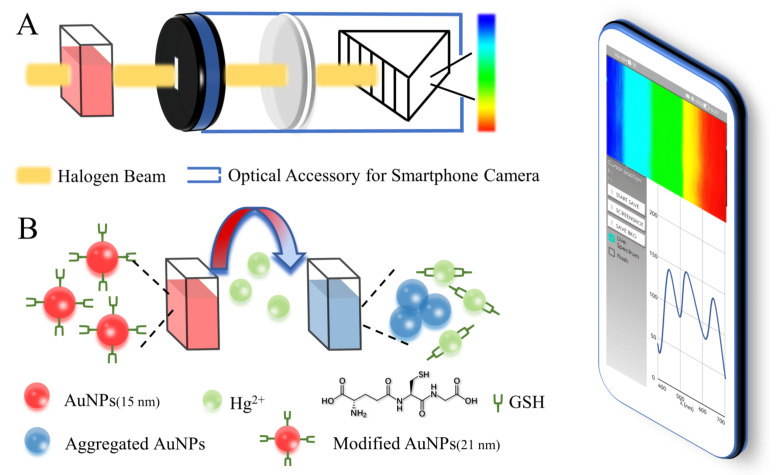
(**A**) Colorimetric biosensor system based on a smartphone; (**B**) schematic illustration of Hg^2+^ detection based on AuNPs-GSH.

Based on the studies referenced above, we constructed a colorimetric biosensor system that uses a smartphone miniature spectrometer as a readout tool (Figure 1A) in this work. As a part of evaluating the sensor, we used a monochromatic diode and a gold nanoparticles (AuNPs) solution to evaluate the accuracy and stability of the proposed system. We also used gold nanoparticles-glutathione (AuNPs-GSH) conjugate as a detection probe to test the practical applicability of the proposed sensor (Figure 1B). According to the three classifications of drinking water in China’s national standard (GB19298-2014, GB5749-2006) and an essential source of drinking water, we selected natural mineral water, pure water, tap water, and river water as actual samples for the spiking experiment and obtained satisfactory results.

## 2. Experimental Section

### 2.1. Materials and Instruments

The details have been listed in Supporting Information.

### 2.2. Preparation of AuNPs and AuNPs-GSH

Gold nanoparticles (AuNPs) were prepared using the trisodium citrate reduction method established by Frens in 1973 [35]. We added 85.8 mL of ultrapure water to a three-necked round-bottomed flask, followed by 4.2 mL of chloroauric acid solution (1%), and then the solution was heated to 120 °C and stirred under magnetic reflux until boiling. Then, 10 mL of trisodium citrate solution (1%) was added rapidly, and the color of the solution turned red within 1 min. We then continued to boil the solution for 20 min and subsequently cooled it to room temperature naturally to obtain a wine-red AuNPs solution. This solution was then stored at 4 °C for later use within a one-year period.

Then, 30 μL of the GSH solution (25 mg/mL) was added to 6 mL of the AuNPs solution. The mixture was placed on a magnetic stirrer and was stirred at room temperature for 2 h to modify the AuNPs completely using GSH via the Au-S bond. The solution was then centrifuged at high speed (30 min, 12,000 rpm, 4 °C) to remove the supernatant containing unreacted GSH, and deionized water was added to re-suspend the AuNPs-GSH conjugates. This solution was also stored at 4 °C for later use within a two-month period.

### 2.3. Evaluation of Sensor Capability for Colorimetric Detection

First, a commercial miniature optical fiber spectrometer (Avantes) was used to detect four narrow-band monochromatic diode light sources. The results obtained were then used as the actual wavelength bands for the monochromatic diodes. After a fixed distance was set between each diode and the smartphone, the smartphone was set to detect the diodes at 2 s detection intervals. In addition, the band position and intensity data of the single peaks were recorded to verify the accuracy and stability of the smartphone’s detection performance.

Further detection and absorbance analyses of AuNPs solutions with various concentrations (1, 5, 10, 25, 50, 75, and 100 μM) were performed to verify the colorimetric detection capability of the smartphone. In this experiment, deionized water was used as the background for the absorbance analysis. For comparison and verification, the background and each concentration of AuNPs solution were automatically detected 20 times by an Avantes micro-optical fiber spectrometer and then the average detected values were exported. Under the same conditions, the smartphone performed 20 detections for the same solution and then obtained the average value. In addition, the 1st, 10th, and 20th of 20 detections for each concentration were selected as three parallel experiments of this concentration.

Limit of detection (LOD) was one of the indicators used to evaluate our detection performance. It was calculated by 3 sb/slope (sb: standard deviation of the background, and the slope in the calibration plot).

### 2.4. Practical Application of Sensors to Colorimetric Detection

#### 2.4.1. PH and Response Time Optimization

The pH of the AuNPs-GSH solution was adjusted to values of 3.5, 4.5, 5.5, 6.5, and 7.5. Then, 5 μM and 10 μM Hg^2+^ solutions were added to each of the AuNPs-GSH solutions with different pH values, and the V_Hg2+_:V_AuNPs-GSH_ was 2:1.The spectral data of these solutions were detected at 522 nm and recorded every 3 s using the smartphone, and the detection process lasted for 30 s. Each assay in these experiments was repeated three times. In the analysis, the absorbance data of the solutions at 522 nm were used as indicators of the degree of dispersion or aggregation of the AuNPs.

#### 2.4.2. Detection of Specificity

The AuNPs-GSH solution was mixed with 16 common ions (Ni^2+^, Cd^2+^, Cr^3+^, Hg^2+^, Pb^2+^, Mn^2+^, Cu^2+^, Ca^2+^, Mg^2+^, Zn^2+^, Fe^3+^, CrO_4_^2−^, CO_3_^2−^, SO_4_^2−^, CH_3_COO^−^, and PO_4_^3−^ ions, 5 μM) at a volume ratio of 1:2 and were detected under the same experimental conditions using the proposed sensor, and each detection process was repeated three times. Ten metal ions (Ni^2+^, Cd^2+^, Cr^3+^, Pb^2+^, Mn^2+^, Cu^2+^, CrO_4_^2−^, Ca^2+^, Mg^2+^, Zn^2+^, and Fe^3+^ ions, 0.5 μM) were mixed. The mixture solution with/without Hg^2+^ (0.5 μM) was added to the AuNPs-GSH solution and detected with the same detection method as above.

#### 2.4.3. Quantitative Detection

We added Hg^2+^ solutions with different concentrations (V_AuNPs-GSH_:V_Hg2+_ = 1:2), and then each mixture was incubated for 5 s. To better use the smartphone for detection in this case, the mixtures obtained above were then diluted at a ratio of 1:4 with deionized water. The smartphone’s spectral data were detected and recorded from 400 nm to 700 nm. The relative distances between the light source, the cuvette, and the smartphone were all fixed. The total detection time was approximately 300 s. The detection time refers to the time between the dropwise addition of the first Hg^2+^ solution concentration to the AuNPs-GSH solution and the detection of the final Hg^2+^ solution concentration. The experiment was repeated three times. Among the results, the absorbance data acquired at 522 nm were used to provide the index parameter for the Hg^2+^ content, which also represented the degree of aggregation or dispersion of the AuNPs.

## 3. Results and Discussion

### 3.1. Smartphone Functions of the Sensor

**Figure 2:**
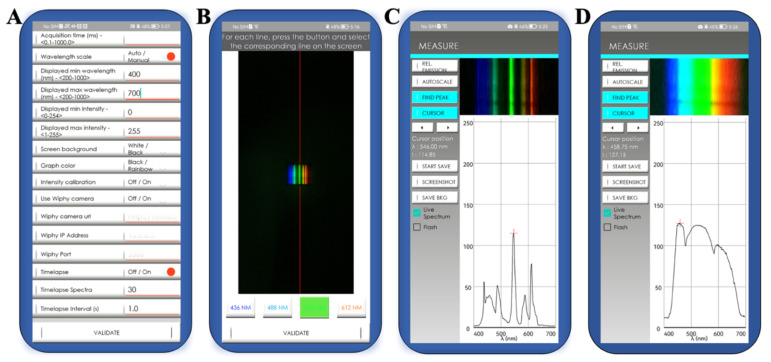
Operating interface of GoSpectro app (Android) on the smartphone. (**A**) Detection parameter settings; (**B**) calibration operation with calibration light source; (**C**) detection of mercury lamp; and (**D**) detection of white light source.

The colorimetric biosensing system constructed in this work used the smartphone camera as a readout tool. The light band image that was formed by the light beam when dispersed by the optical accessories reached the smartphone’s camera. The smartphone then collected the detected spectral data based on the image received (this principle is illustrated in Figure S1, Supplementary Materials). The operational interface of the mobile terminal processing software (GoSpectro app) is shown in Figure 2. The accuracy and stability of the spectrum band data obtained are vital to the detection process. The calibration process can be adjusted using the parameter settings before detection and the calibration light source. After the calibration process is complete, the smartphone software will then match the pixel position and intensity of the received image with the position and intensity of the band. This will enable the formation of the visual and spectral data simultaneously. These data can be read and recorded in real-time. This is true whether the data are acquired for mercury lamps with multiple narrow-band light sources or for halogen lamps with a broader spectrum of wavelengths. We detected mercury lamps to validate that using the above process could obtain available and similar results from different low-configuration smartphones (Table S1 and Figure S2).

### 3.2. Evaluation of Sensor Performance in Colorimetric Detection

#### 3.2.1. Accuracy and Stability of Sensor Detection

**Figure 3:**
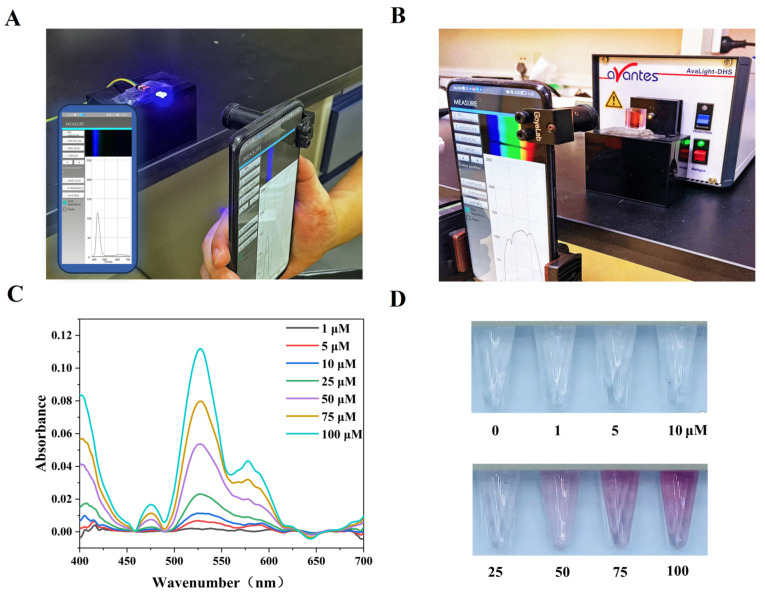
(**A**) Verification of the accuracy and stability of the sensor proposed in this paper via the detection of monochromatic diodes; (**B**) detection of AuNPs solutions via the sensing system; (**C**) absorbance analysis results for AuNPs solutions with different concentrations; (**D**) color changes in the solutions with increasing AuNPs concentrations.

Using a smartphone as a reading device involves two primary abilities: the wavelength band must be detected accurately, and the light intensity detection must remain relatively stable. This part of the experimental process was verified using four monochromatic diodes with different wavelengths (Figure 3A). The results in Table S2 show that the position of the detection peak of the proposed sensor is basically the same as that of the Avantes spectrometer, and the existing sensor error will not affect the detection significantly (0.5 nm).

These results also verified that the calibration operation performed before detection was effective. Otherwise, the calibration would cause the image pixels to fail to correspond to the band’s position, and the detection peak would have been shifted. Furthermore, the fluctuations in the light intensity data for the detection peak are minimal, thus indicating that the impact of the system error is limited. This illustrates the good repeatability of the detection operation.

The cooperation between the smartphone camera and the optical accessories also indicates that the sensing system can provide an accurate and stable detection performance after a standard calibration operation.

#### 3.2.2. Sensitivity Analysis of Sensor Detection

For colorimetric detection applications, it is imperative that the sensor be highly sensitive to color. It is possible to change the color of the solution under test gradually, from colorless to wine red, by varying the AuNPs concentration. Therefore, we analyzed the detection and absorbance of AuNPs solutions with different concentrations to verify the smartphone’s detection performance (Figure 3B). As shown in Figure 3C, the absorbance analysis indicated that the AuNPs appeared to have a prominent absorption peak at approximately 522 nm due to the 15 nm AuNPs. In addition, within the 625–650 nm range, there is a fluorescence effect that results in the appearance of a negative absorbance peak for this band range. These two characteristic peaks showed good repeatability in multiple experiments, and the Avantes spectrometer under the same experimental conditions verified this phenomenon as well.

We performed linear regression analyses on the absorbance data acquired at 522 nm from both the smartphone and the Avantes spectrometer. These analyses evaluated the detection capability of the smartphone. Both detection devices showed excellent linear relationships between the concentration and the absorbance of the AuNPs within the detection range from 1 μM to 100 μM (Figure S3). The correlation coefficients of the two devices were R^2^ = 0.99922 and R^2^ = 0.99975, respectively, and their slopes differed by only 0.0002. Additionally, the limit of detection (LOD, 3 sb/slope) for AuNPs of the smartphone reached 0.14 μM (Figure S3A). These results showed that the sensing system provided a detection capability comparable to that of a commercial spectrometer over an extensive range of variations of the same color. It was noted that even when the AuNPs solution appeared to the naked eye as having little to no color, or when the colors of the solutions were so close to each other that it was difficult to differentiate between them (Figure 3D), the smartphone could detect the gradient successfully. This provides an intuitive illustration of the sensitivity and conveys that the smartphone camera is highly suited to the capture of color changes, thus meeting the colorimetric application requirements.

### 3.3. Practical Application of the Sensors to Hg^2+^ Detection

AuNPs are used widely because of their excellent optical properties. We selected the detection performance for Hg^2+^ sensing based on GSH-modified AuNPs to evaluate the practical applicability of the sensor. This allows the sensor to work under more complex color change conditions to ensure that comprehensive performance testing can be obtained during practical applications. The detection process based on use of AuNPs as probes can also reflect the scalability of the sensor in colorimetric detection.

#### 3.3.1. Principle and Characterization of Hg^2+^ Detection by AuNPs-GSH

AuNPs have a high affinity for biological thiols, and when molecules containing thiol groups are added to the AuNPs solution, they will bind rapidly to the surfaces of the AuNPs [36]. When compared with AuNPs and other metal ions, Hg^2+^ has higher thiophilic properties [37]. Based on this principle, several studies have been performed that have led to the aggregation of AuNPs, which results in a color change from red to blue [38,39,40] or causes aggregated AuNPs to achieve an anti-aggregation effect, which in turn causes the solution color to change from blue to red as the particles are re-dispersed [41,42,43]. This enables the detection of Hg^2+^ or biothiols using AuNPs. Based on the studies described above, trisodium citrate-reduced AuNPs (citrate-AuNPs) were modified with GSH via Au-S bonds at room temperature to form a stable AuNPs-GSH probe. When a specific volume of the Hg^2+^ solution was added to the AuNPs-GSH solution, the extremely high affinity of -SH toward Hg^2+^ triggered the breakage of Au-S bonds on surfaces of AuNPs, causing GSH to fall from Au surfaces and then form a GSH-Hg-GSH complex with Hg^2+^, thus destabilizing the AuNPs and causing aggregation and color changing [44]. When the Hg^2+^ concentration increases, the AuNPs solution follows a red-purple-blue color change pattern, which also places higher requirements on the tests of the proposed sensor’s detection performance.

**Figure 4:**
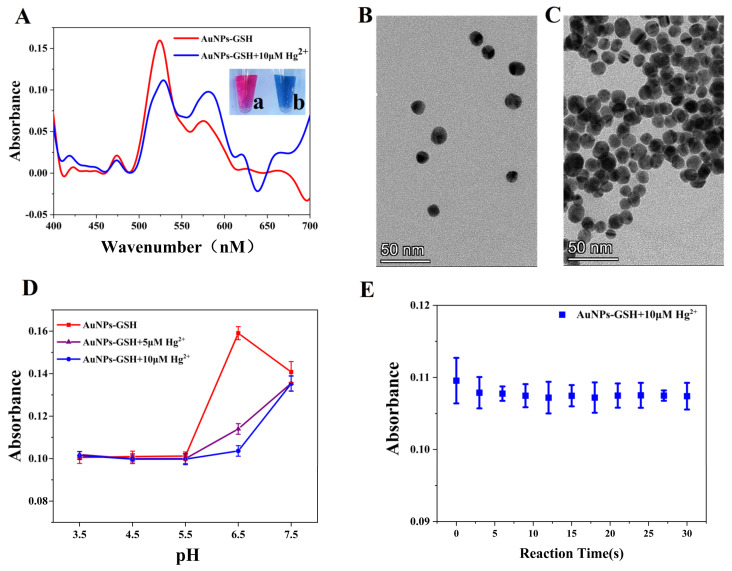
(**A**) Characterization of the absorption spectra of the AuNPs−GSH solution using the proposed sensor (a) without Hg^2+^ and (b) with 10 μM of Hg^2+^; (**B**,**C**) TEM images of the AuNPs−GSH solution before and after addition of Hg^2+^; (**D**) absorbance analysis results before and after addition of Hg^2+^ to the AuNPs−GSH solution under different acid−base environmental conditions; (**E**) changes in absorbance over time after addition of Hg^2+^ to the AuNPs−GSH solution.

To verify the capability of the practical smartphone detection process and the feasibility of the proposed method, we first characterized the AuNPs-GSH solution using the sensing system presented in this paper (Figure 4). The AuNPs-GSH absorption peak is located near 522 nm. The addition of a high Hg^2+^ concentration causes the AuNPs-GSH solution to aggregate and rapidly change from red to blue. We also observed the process of the AuNPs-GSH solution before and after aggregation by transmission electron microscopy (TEM). Figure 4B showed that the prepared AuNPs-GSH had a good dispersion, but when a specific concentration of the Hg^2+^ solution was added to the AuNPs-GSH solution, the AuNPs then aggregated (Figure 4C). The absorption peak in Figure 4A showed a significant shift to the right with the addition of Hg^2+^, which is related to the red-shift phenomenon caused by the larger overall diameter of the aggregated AuNPs. Further, we used dynamic light scattering (DLS) to characterize the size distribution changes of AuNPs, AuNPs-GSH, and AuNPs-GSH+Hg^2+^. The results (Figure S4) showed that the average size of AuNPs is about 15 nm. The hydrodynamic size of AuNPs-GSH increased to about 21 nm after the conjugation of GSH on the surface of AuNPs. We also validated the aggregation process using DLS, which revealed that the hydrodynamic size of AuNPs was dramatically enlarged relative to the AuNPs-GSH when Hg^2+^ was introduced. The zeta potential also was tested as well. Compared with AuNPs, the zeta potential of AuNPs-GSH changed from −44.3 to −26.6 mV, which suggested that GSH has been successfully modified on the surfaces of AuNPs, and it changed to −1.56 mV after adding Hg^2+^. These characterization processes verify that the AuNPs-GSH-based probe has the potential to detect Hg^2+^, while the accurate detection of the red-shift phenomenon also illustrates the excellent performance of the proposed sensor.

#### 3.3.2. Optimization of the Experimental Conditions

To remove the influence of certain factors in the experiment of smartphone detection performance and to provide a better reflection for the practical application of the sensor proposed in this paper, we analyzed and optimized the pH value of the solution and the response time to the AuNPs-GSH solution with Hg^2+^. Figure 4D showed that the AuNPs were extremely unstable in an acidic environment and exhibited low absorbance. From a combination of the absorbance values with the electron microscope images (Figure 4A,D), it can be inferred that the AuNPs were aggregated to a high degree at this time, which also means that even if Hg^2+^ was added to the solution, no significant change would be observed. According to the results of this analysis, the detection should be performed in a weak acid environment. Because the reaction of the solution tends to become stable within approximately 5 s of adding Hg^2+^ (Figure 4E), the incubation time for the probe and target was set at 5 s. In this section, the smartphone detection results demonstrated the device’s rapid-detection capability and reflected the aggregation of the AuNPs accurately.

#### 3.3.3. System-Specific Analysis and Quantitative Detection

**Figure 5:**
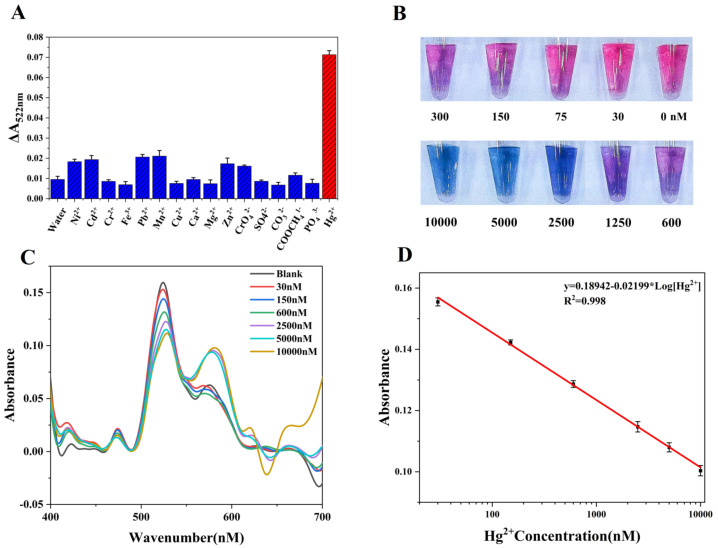
(**A**) Absorbance at 522 nm of AuNPs−GSH solutions when mixed with 16 different metal ions; (**B**) color changes caused by the addition of different concentrations of Hg^2+^ to the AuNPs−GSH solution; (**C**) absorption spectra of the solution in (**B**) when detected by the proposed sensor; (**D**) linear relationship between absorbance at 522 nm and the Hg^2+^ concentration.

The specific selection of AuNPs-GSH is essential to guarantee the effective detection of Hg^2+^ by the proposed sensor. In the experiments, we adjusted the volume ratio of the AuNPs-GSH solution to 16 common ions solutions (1:2) appropriately, and the color changed dramatically when Hg^2+^ was dropped into this solution. Additionally, the change in this solution was most obvious in the 5 min following the addition of Hg^2+^ (Figure S5). To better illustrate this feature, we used the smartphone to detect the absorbance at 522 nm after the AuNPs-GSH solution was mixed with common ions (Figure S6), as well as the value of the resulting absorbance change (Figure 5A). When the AuNPs-GSH solution was mixed with deionized water as a control group, it was found that Hg^2+^ produced a very prominent response, whereas the influence of adding other ions to the solution was similar to the control group. To a certain extent, this also showed that interference from the other ions is very limited at the volume ratio of 1:2. Under the same conditions, when we added the mixture solution containing 10 metal ions (excluding Hg^2+^) to AuNPs-GSH, there was no obvious interference. However, the mixture solution containing Hg^2+^ caused AuNPs aggregation (Figure S7), which showed that our method still had good selectivity when ions competed with each other.

Based on the work described above, it was determined that the colorimetric biosensor of the smartphone is capable of detecting Hg^2+^ in water by AuNPs-GSH. We then evaluated the quantitative detection effect of the sensor under optimized conditions. When the concentration of Hg^2+^ increased, the aggregation of the AuNPs in the solution also increased, which means that the absorbance of the solution near 522 nm decreased gradually as the Hg^2+^ concentration increased from 0 to 1 × 10^4^ nM (Figure S8), and this change was accompanied by a red-shift phenomenon (Figure 5C). The LOD (3 sb/slope) of Hg^2+^ is 1.2 nM/0.24 ppb (Figure 5D), which is well under the international community’s limit values of 5 nM (1 ppb) and 10 nM (2 ppb) for Hg^2+^ in drinking water. The change in the Hg^2+^ concentration and absorbance showed a good linear relationship within the detection range from 30 nM to 1 × 10^4^ nM (Figure 5D), which indicates that the colorimetric biosensor system, based on the use of a smartphone as a miniature spectrometer as proposed in this paper, could be used effectively to perform quantitative Hg^2+^ detection. Furthermore, rapid detection and high sensitivity were both achieved within an extensive color variation range (red-purple-blue), even when the solutions were difficult to distinguish with the naked eye (Figure 5B). This verified the reliability of the sensor’s colorimetric detection performance in practical applications.

#### 3.3.4. Spiking Experiment in Actual Samples

When establishing the calibration model, the sample preparation solution was standard purified water prepared in the laboratory. However, the water-quality parameters for natural water bodies are not the same as for standard purified water. When the configuration solution is from a natural water body, the substances in the water may cause results to be inaccurate, so the detection and evaluation of actual water samples is essential. According to the three classifications of drinking water in China’s national standard (GB19298-2014, GB5749-2006), we selected natural mineral water, pure water, and tap water as actual samples for the spiking experiment.

Analytical results in Table 1 showed that the recoveries varied from 97.43% to 102.98% in the spiked Hg^2+^ samples. The recovery values indicated that our proposed method could be used for highly accurate Hg^2+^ detection in actual samples from different drinking waters.

In addition, river water is an essential part of the ecological system and is an important source of drinking water, so we have also listed it as our test object. Due to the complex composition of river water, we left the samples standing overnight and diluted them five times with deionized water. For comparison, the inductively coupled plasma-mass spectrometry (ICP-MS) was also used to detect the spiked samples. As listed in Table S3, the results of the presented method were similar to those of ICP-MS, with the recoveries varying from 99.76% to 115.88%, which indicated that the proposed method showed excellent potential for detecting Hg^2+^ in complex, river water samples.

Furthermore, we compared the performance of the proposed strategy with those of other sensors for Hg^2+^ detection, based on AuNPs and on the use of a professional spectrometer or smartphone as the readout method, as shown in Table 2. First, we could obtain a detection limit with the same order of magnitude as those of precision instruments based on the Hg^2+^ detection of the smartphone. Second, when compared with the other methods presented, the Hg^2+^ detection method proposed in this paper showed a comparable linear range and faster response time. Third, combined with Table S1, we find that our method could work well with most cheap, mid- to low-end phones currently on the market. Additionally, compared to other smartphone-based methods, we can perform a more detailed analysis that incorporates spectral information rather than solely the data of color. Finally, through practical application to Hg^2+^ detection and by comparison with related work from other researchers, this paper demonstrated that the colorimetric biosensor with a smartphone as a low-cost micro-spectrometer has great potential for future applications, and that this sensor can perform colorimetric detection accurately, effectively, and stably within the visible light range.

## 4. Conclusions

In this work, we built a colorimetric micro-spectrometer system that used a smartphone as a readout tool for the detection of Hg^2+^ in drinking water. The mobile terminal of the sensor processed the image received using a combination of the smartphone’s camera and optical accessory, and ultimately achieved the desired spectral detection effect. In the evaluation of smartphone detection performance, effective detection of both the target light source and the liquid being tested were achieved, and the detection limit of AuNPs reached 0.14 μM. Additionally, when the samples were colorless or very similar in color, the smartphone detection method showed high sensitivity in capturing color changes. We also used the sensor to detect more complex sample-color changes. Therefore, we used AuNPs-GSH as a detection probe material to detect Hg^2+^ in water, and obtained a LOD of 1.2 nM. This method was also applied successfully in actual samples of natural mineral water, pure water, tap water, and river water, with a recovery rate range of 97.4~115.9%. Following a series of performance evaluations and practical applications, it was concluded that the sensor proposed in this paper could use the smartphone as a micro-spectrometer to collect the spectral signals of AuNPs and perform accurate, stable, and fast colorimetric detection with a highly sensitive readout capability. This sensor has tremendous potential for future application in low-cost biological colorimetric rapid detection.

## Figures and Tables

**Table 1:** Analytical results of samples.

Sample	Added (nM)	Found (nM)	Recovery (%)	RSD (%, *n* = 3)
Tap water	75	77.23	102.98	3.25
	300	299.07	99.68	1.43
	1250	1267.68	101.41	1.98
	5000	5123.52	102.65	2.02
Mineral water	75	75.575	100.76	3.58
	300	295.61	98.54	3.23
	1250	1217.87	97.43	2.61
	5000	4938.94	98.78	2.79
Pure water	75	74.91	100.14	1.06
	300	295.82	98.61	1.70
	1250	1277.33	102.18	1.86
	5000	4958.97	99.18	1.15

**Table 2:** Comparison between the practical application effect of the proposed colorimetric biosensor to Hg^2+^ detection and the results of previously reported works.

Materials	Incubation Time(s)	Tool *	Linear Rangen(M)	Sample	LOD for Hg^2+^	Ref.
AuNPs-MBT	300	S	50–10^3^	Lake water	6.0 nM/1.20 ppb	[45]
AuNPs-CTAB	1800	S	20–10^3^	Tap water	11.9 nM/2.39 ppb	[46]
AuNPs-MSA	300	S	10–10^4^	Tap water	4.8 nM/0.96 ppb	[47]
AuNPs-AA	300	S	9–1.27 × 10^4^	Tap water	8.8 nM/1.76 ppb	[48]
AuNPs-APTES	1200	S	15–92	River water	10 nM/2.01 ppb	[49]
AuNPs-DETL	900	S	100–5 × 10^3^	River water	24.0 nM/4.81 ppb	[37]
AuNPs-DTT	480	P	54–267	Rain water	17.0 nM/3.40 ppb	[50]
AuNPs-H_2_O_2_	1800	P	100–10^4^	Lake water	40.0 nM/8.02 ppb	[51]
AuNPs-GSH	5	P	30–10^3^	River water	1.2 nM/0.24 ppb	**T** **his work**

* Readout tool type. S: Spectrophotometer, P: Phone.

## Data Availability

Not applicable.

## Supplementary Materials

The following supporting information can be downloaded at: https://www.mdpi.com/article/10.3390/bios12111017/s1, Figure S1: The light source accessory (GoSpectro) is installed before the cell phone camera. The light beam A is first passed through a collimating lens B through a slit and is divided by a diffraction grating and a trigonal prism C to form a visible light band, which is received by the cell phone camera; Table S1: Smartphones related information; Figure S2: Detection of mercury lamp sources. (A) The influence of different exposure times on Huawei smartphone. (B) The results of the three phones can be very close after the calibration and adjustment of the exposure time; Table S2: Performance verification results after the smartphone was debugged, where each monochromatic diode was detected 20 times during the experiment at 2 s intervals; Figure S3: (A) Quantitative detection of AuNPs by the sensor in this paper; (B) Quantitative detection of AuNPs by commercial Avantes spectrometer; Figure S4: Size distribution and Zeta potential of AuNPs, AuNPs-GSH, and AuNPs-GSH+Hg^2+^; Figure S5: Influence of various metal ions on the color of AuNPs-GSH solution; Figure S6: Influence of various metal ions on the absorbance of AuNPs-GSH solution; Figure S7: The change of absorbance in AuNPs-GSH solution after adding deionized water and metal ions mixture solution with/without Hg^2+^ (0.5 μM), respectively. A, AuNPs. M, metal ions mixture solution (Ni^2+^, Cd^2+^, Cr^3+^, Pb^2+^, Mn^2+^, Cu^2+^, CrO_4_^2−^, Ca^2+^, Mg^2+^, Zn^2+^, and Fe^3+^ ions, 0.5 μM); Figure S8: The change in absorbance corresponds to the concentration of Hg^2+^; Table S3: Determination of Hg^2+^ in river water by the proposed method.
