# Supplementary material for: A Real-Time Detection Method of Hg2+ in Drinking Water via Portable Biosensor: Using a Smartphone as a Low-Cost Micro-Spectrometer to Read the Colorimetric Signals
Source: Biosensors (Basel). 2022 Nov 14;12(11):1017. doi: 10.3390/bios12111017 (PMC9688040; doi:10.3390/bios12111017)
Supplement: Supplementary file 1 [file biosensors-12-01017-s001.zip › biosensors-1973659-supplementary.pdf]

Supplementary Material

A Real-Time Detection Method of Hg^{2+} in Drinking Water Via Portable Biosensor: Using a Smartphone as a Low-Cost Micro-Spectrometer to Read the Colorimetric Signals

Yifan Gu, Leizi Jiao, Fengjing Cao, Xinchao Liu, Yunhai Zhou, Chongshan Yang, Zhen Gao, Mengjie Zhang, Peng Lin, Yuxing Han and Daming Dong

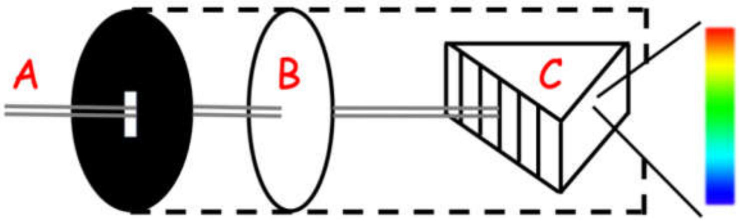

Figure S1. The light source accessory (GoSpectro) is installed before the cell phone camera. The light beam A is first passed through a collimating lens B through a slit and is divided by a diffraction grating and a trigonal prism C to form a visible light band, which is received by the cell phone camera.

Table S1. Smartphones related information.

Brand	Model	CPU	Launch (Year)	Price (US $)
Huawei	STK-l00	Hisilicon Kirin-710F	2019	195
Xiaomi	MI6X	Snapdragon 660	2018	124
VIVO	X6plusD	MediaTek MT6752	2015	325

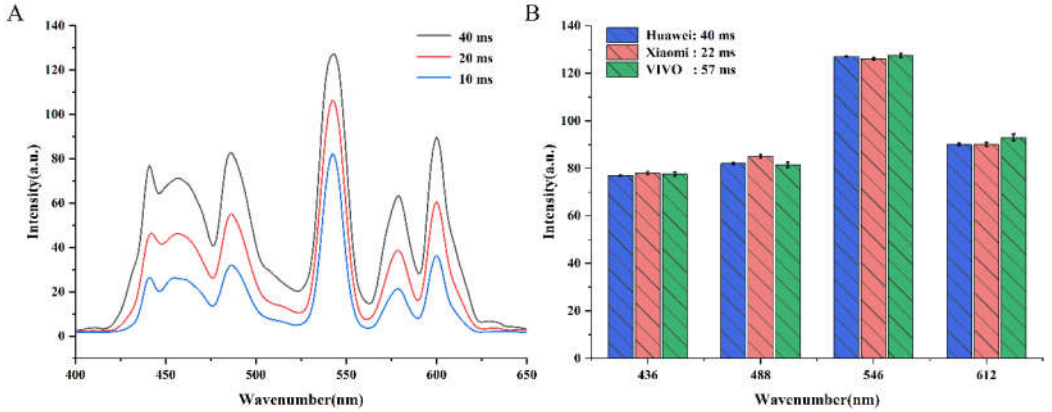

Figure S2. Detection of mercury lamp sources. (**A**) The influence of different exposure times on Huawei smartphone. (**B**) The results of the three phones can be very close after the calibration and adjustment of the exposure time.

Table S2. Performance verification results after the smartphone was debugged, where each monochromatic diode was detected 20 times during the experiment at 2 s intervals.

NO.	Actual Wavelength (nm)	Detection Wavelength (nm)	Standard Deviation (n = 20)	Detection Light Intensity (a.u.)	Standard Deviation (n = 20)
#1	444	444.30	0.78	117.69	0.28
#2	552	552.05	0.61	112.80	0.16
#3	642	641.64	0.81	138.41	0.41
#4	661	661.08	0.54	198.98	0.43

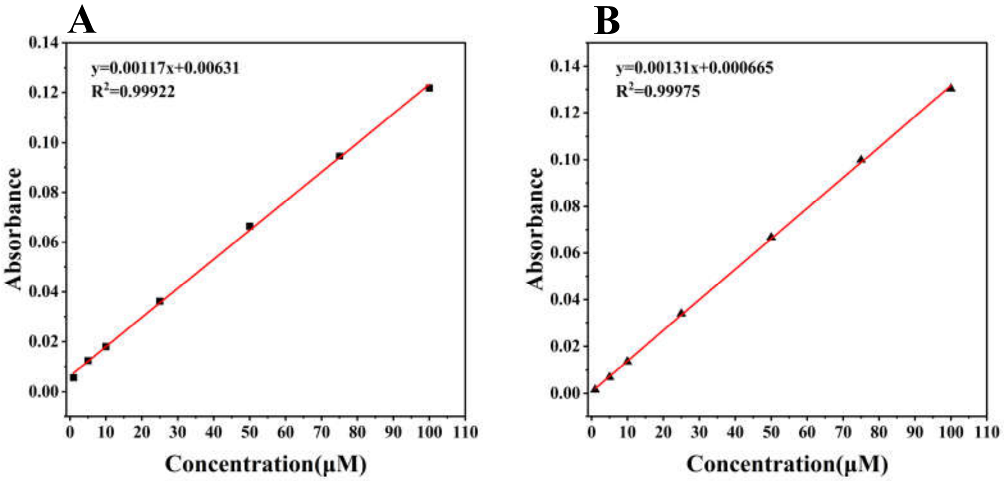

Figure S3. (**A**) Quantitative detection of AuNPs by the sensor in this paper; (**B**) Quantitative detection of AuNPs by commercial Avantes spectrometer.

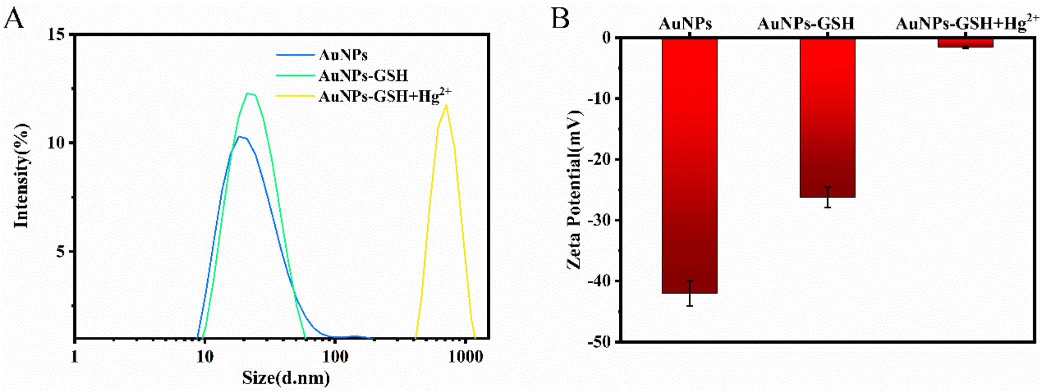

Figure S4. Size distribution and Zeta potential of AuNPs, AuNPs-GSH, and AuNPs-GSH+Hg^{2+}.

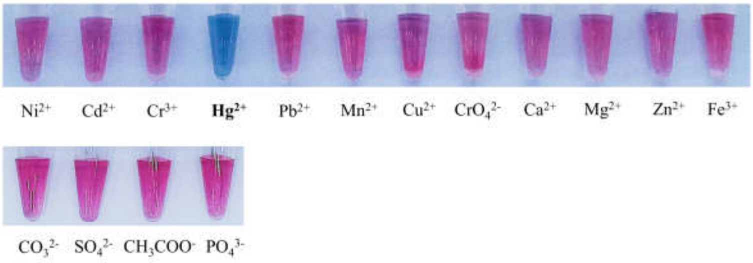

Figure S5. Influence of various metal ions on the color of AuNPs-GSH solution.

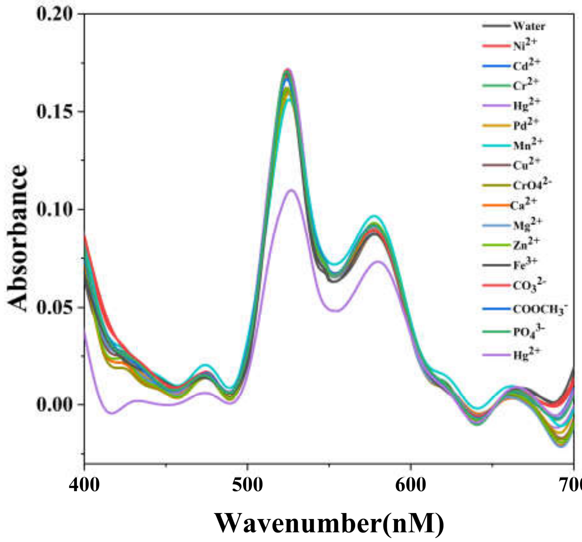

Figure S6. Influence of various metal ions on the absorbance of AuNPs-GSH solution.

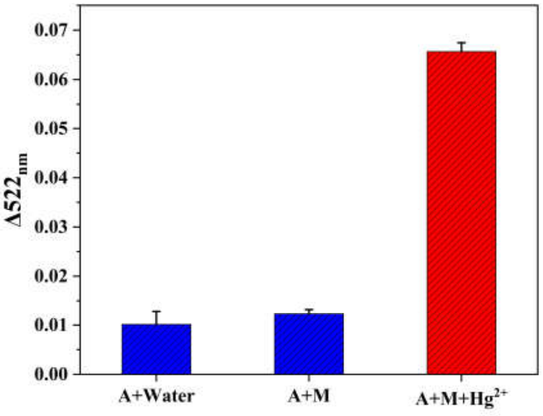

Figure S7. The change of absorbance in AuNPs-GSH solution after adding deionized water and metal ions mixture solution with/without Hg^{2+} (0.5 μM), respectively. A, AuNPs. M, metal ions mixture solution (Ni^{2+}, Cd^{2+}, Cr^{3+}, Pb^{2+}, Mn^{2+}, Cu^{2+}, CrO_4^{2-}, Ca^{2+}, Mg^{2+}, Zn^{2+}, and Fe^{3+} ions, 0.5 μM).

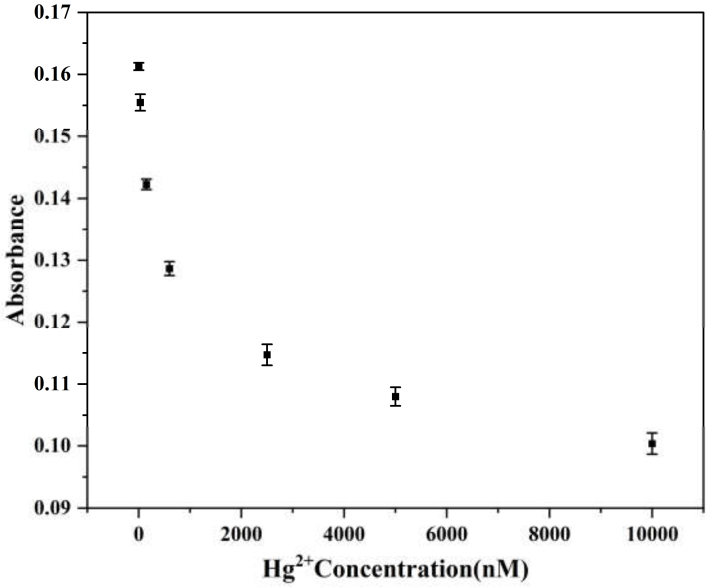

Figure S8. The change in absorbance corresponds to the concentration of Hg^{2+}.

S1. Spiking experiment in river water samples.

Herein we selected river water as actual samples for the spiking experiment. We left the samples standing overnight and diluted them five times with deionized water; Hg^{2+} was introduced to the samples at three concentrations of 500, 2500 and 5000 nM. For comparison, the inductively coupled plasma-mass spectrometry (ICP-MS) method was also used to detect the spiked samples. As listed in Table S3, the results of the presented method were similar to those of ICP-MS, which indicated that the proposed method showed excellent applied prospects for detecting Hg^{2+} in complex river water samples.

Table S3. Determination of Hg^{2+} in river water by the proposed method.

Sample	Added (nM)	Found (nM)	RSD (%, n = 3)	Recovery (%)	ICP-MS (nM)
#1	0	<LOD	—	—	0.51
#2	500	579.43	0.99	115.88	538.66
#3	2500	2494.05	6.48	99.76	2397.05
#4	5000	5074.47	4.20	101.49	4764.32

S2. Materials and Instruments.

All reagents and solutions were analytical grade and were ready to use. Chloroauric acid ($HAuCl_4$) and trisodium citrate were acquired from Sigma, USA. Glutathione (GSH) was acquired from Shanghai McLean Biochemical Technology Co., Ltd. Ni^{2+}, Cd^{2+}, Cr^{3+}, Hg^{2+}, Pb^{2+}, Mn^{2+}, Cu^{2+}, CrO_4^{2-}, Ca^{2+}, Mg^{2+}, Zn^{2+}, Fe^{3+}, and other metal standard solutions were obtained from Beijing Northern Weiye Metrology Technology Research Institute. The experimental water was deionized water. and the actual water samples used were river water (from the Beijing Jingmi Diversion Canal). The instruments and equipment used include the Talos F200X G2 transmission electron microscope (Thermo Fisher Scientific, USA). The AvaSpec-ULS2048 micro-fiber spectrometer and AvaLight-DHS light source (Beijing Avantes

Technology Co., Ltd.). A 1 mL four-sided transparent quartz cuvette with an optical path length of 10 mm (Yixing Spectrum Optical Components Co., Ltd.). The MYP11-2A magnetic stirrer (Shanghai Meiyingpu Instrument Manufacturing Co., Ltd.). The TGL-15B desktop high-speed centrifugal machine (Shanghai Anting Scientific Instrument Factory). The KQ-250DE CNC ultrasonic cleaner (Kunshan Ultrasonic Instrument Co., Ltd.). A Huawei STK-AL00 smartphone (Huawei Technology Co., Ltd.). GoSpectro smartphone camera optical accessories (GoyaLab Team, France). And a monochromatic narrow-band diode (Shenzhen Zhongshan Shuanghong Electronic Technology Co., Ltd.).
